# Hereditary orotic aciduria identified by newborn screening

**DOI:** 10.3389/fgene.2023.1135267

**Published:** 2023-03-14

**Authors:** Orna Staretz-Chacham, Nadirah S. Damseh, Suha Daas, Nasser Abu Salah, Yair Anikster, Ortal Barel, Elena Dumin, Aviva Fattal-Valevski, Tzipora C. Falik-Zaccai, Eli Hershkovitz, Sagi Josefsberg, Yuval Landau, Tally Lerman-Sagie, Hanna Mandel, Rachel Rock, Nira Rostami, Talya Saraf-Levy, Nava Shaul Lotan, Ronen Spiegel, Galit Tal, Igor Ulanovsky, Yael Wilnai, Stanley H. Korman, Shlomo Almashanu

**Affiliations:** ^1^ Metabolic Clinic, Pediatric Division, Soroka University Medical Center, Ben Gurion University, Beer- Sheva, Israel; ^2^ Faculty of Health Sciences, Ben-Gurion University, Beer Sheva, Israel; ^3^ Institute for Rare Diseases, Soroka University Medical Center, Ben Gurion University, Beer- Sheva, Israel; ^4^ Faculty of Medicine, Al-Quds University, Palestinian National Authority, Abu Deis, Palestine; ^5^ National Newborn Screening Program, Public Health Services, Ministry of Health, Ramat-Gan, Israel; ^6^ Department of Neonatology, Red Crescent Society Hospital, Jerusalem, Israel; ^7^ School of Medicine, Hebrew University School of Medicine, Jerusalem, Israel; ^8^ Sackler School of Medicine, Tel Aviv University, Tel-Aviv, Israel; ^9^ Metabolic Disease Unit, Sheba Medical Center Tel-Hashomer, Edmond and Lily Safra Children’s Hospital, Ramat Gan, Israel; ^10^ Genomics Unit, The Center for Cancer Research, Sheba Medical Center, Ramat Gan, Israel; ^11^ Metabolic Laboratory, Sheba Medical Center, Ramat Gan, Israel; ^12^ Ruth and Bruce Rappaport Faculty of Medicine, Technion-Israel Institute of Technology, Haifa, Israel; ^13^ Tel Aviv Sourasky Medical Center, Dana Children Hospital, Pediatric Neurology Institute, Tel Aviv, Israel; ^14^ Galilee Medical Center, Institute of Human Genetics, Naharia, Israel; ^15^ The Azrieli Faculty of Medicine, Bar Ilan, Safed, Israel; ^16^ Pediatric D Department, Soroka Medical Center, Beer Sheva, Israel; ^17^ Kaplan Medical Center, Genetics Institute, Rehovot, Israel; ^18^ Metabolic Disease Unit, Schneider Children’s Medical Center, Petah Tikva, Israel; ^19^ Magen Center for Rare Diseases-Metabolic, Neurogenetic, Wolfson Medical Center, Holon, Israel; ^20^ Metabolic Unit, Department of Genetics, Rebecca Sieff Hospital, Safed, Israel; ^21^ Department of Genetics, Hadassah-Hebrew University Medical Center, Jerusalem, Israel; ^22^ Department of Pediatrics B, Metabolic Service, Emek Medical Center, Afula, Israel; ^23^ Emek Medical Center, Institute for Rare Diseases, Afula, Israel; ^24^ Rambam Medical Center, Metabolic Clinic, Ruth Rappaport Children’s Hospital, Haifa, Israel; ^25^ Tel Aviv Sourasky Medical Center, Genetic Institute, Tel Aviv, Israel; ^26^ Shaare Zedek Medical Center, Wilf Children’s Hospital, Jerusalem, Israel

**Keywords:** newborn screening (NBS), hereditary orotic aciduria, uridine monophosphate synthase, orotic acid, megaloblastic anemia, neurodevelopmental disability

## Abstract

**Introduction:** Hereditary orotic aciduria is an extremely rare, autosomal recessive disease caused by deficiency of uridine monophosphate synthase. Untreated, affected individuals may develop refractory megaloblastic anemia, neurodevelopmental disabilities, and crystalluria. Newborn screening has the potential to identify and enable treatment of affected individuals before they become significantly ill.

**Methods:** Measuring orotic acid as part of expanded newborn screening using flow injection analysis tandem mass spectrometry.

**Results:** Since the addition of orotic acid measurement to the Israeli routine newborn screening program, 1,492,439 neonates have been screened. The screen has identified ten Muslim Arab newborns that remain asymptomatic so far, with DBS orotic acid elevated up to 10 times the upper reference limit. Urine organic acid testing confirmed the presence of orotic aciduria along with homozygous variations in the *UMPS* gene.

**Conclusion:** Newborn screening measuring of orotic acid, now integrated into the routine tandem mass spectrometry panel, is capable of identifying neonates with hereditary orotic aciduria.

## 1 Introduction

Hereditary orotic aciduria is an autosomal recessive disease caused by deficiency of the uridine monophosphate synthase (UMPS) enzyme which catalyzes the last step in pyrimidine biosynthesis in mammals (McClard et al., 1980) (EC 4.1.1.23). This bifunctional homodimeric enzyme is encoded by the *UMPS* gene (Suchi et al., 1997) (MIM 613891) and harbors two functions: an orotate phosphoribosyltransferase function (OPRT, EC 2.4.2.10) located in the 214 N-terminal amino acids, and an orotidylic decarboxylase (ODC, EC 4.1.1.23) function located in the 258 C-terminal amino acids (Suttle et al., 1988). Biochemically, the OPRT activity ribosylates orotate to become orotidine monophosphate, while the ODC activity decarboxylates orotidine monophosphate to become uridine monophosphate. Therefore, defects in the UMPS enzyme can lead to a build-up of orotate and/or orotidine monophosphate (OMP) in cells (Bailey, 2009).

Hereditary orotic aciduria was first described in 1959 in an infant presenting with refractory megaloblastic anemia and excretion of orotic acid (Huguley et al., 1959). The disease typically presents in early infancy with megaloblastic anemia, and may be treated with a pyrimidine analog (Uridine triacetate). Later symptoms such as growth retardation, developmental delay and intellectual disability may develop if left untreated (Wortmann et al., 2017). Hematologic malfunction, such as leukopenia, neutropenia, and defective cell-mediated immune deficiency, has also been reported (Girot et al., 1983; Wortmann et al., 2017). In additional, orotic aciduria with subsequent orotate crystalluria has occasionally resulted in urinary obstruction in affected individuals later in life (Bailey, 2009). The most recent case reported was a 17-year-old Emirati girl born to a consanguineous couple reported to have a complicated medical history since early infancy. She presented with unexplained megaloblastic bone marrow, immunodeficiency in form of recurrent infections, epilepsy, developmental delay and crystalluria. The patient showed clinical, hematologic, and biochemical improvement after being treated with uridine triacetate (Al Absi et al., 2021).

Three subtypes of hereditary orotic aciduria have been reported in the literature, all caused by deficiencies in UMPS. Subtype I involves a defect of both OPRT and ODC functions, and subtype II involves a defect in ODC only (Fox et al., 1973). These two biochemical subtypes are clinically indistinguishable, both presenting with megaloblastic anemia, orotic aciduria, and growth and developmental abnormalities (Fox et al., 1973). In contrast, subtype III, resulting also from a biochemical defect in ODC, has been reported in only 2 cases, which presented with orotic aciduria but without megaloblastic anemia (OAWA). Since the report of these cases was prior to the molecular era, these two cases may be simply carriers for the disease (Tubergen et al., 1969; Bailey, 2009; Wortmann et al., 2017). In addition, heterozygosity for *UMPS* variants was recently found to be associated with mild asymptomatic orotic aciduria [OMIM#258900] (Robinson et al., 1984; Wortmann et al., 2017).

Since 2014, and as part of the expanded newborn screening (NBS) program in Israel, orotic acid, and citrulline have been measured in dried blood spots (DBS) for the detection of ornithine transcarbamylase deficiency (OTCD) as a core condition (Staretz-Chacham et al., 2021). Inadvertently, orotic acid as a newborn screening disease biomarker has also led to the identification of hereditary orotic aciduria. During this period, ten neonates have been identified with elevated orotic acid and later found to carry homozygous variants in the *UMPS* gene.

In this study, we report the first cohort of patients identified through newborn screening with hereditary orotic aciduria and presenting with isolated asymptomatic orotic aciduria.

## 2 Materials and methods

The Newborn Screening Program in Israel is a national effort, and all samples are transferred to, handled, and analyzed at a single laboratory. On average, results are reported on the fourth day of life. The clinical data were collected at real time as part of the routine newborn screening and all parents were consented at the referral follow-up clinics.

The National Newborn Screening Program collaborates with all metabolic clinics round the state. These clinics receive referrals of babies with positive newborn screening. A referral of any positive NBS for a metabolic disorder includes the option of rapid confirmatory molecular testing (fresh blood sample in an EDTA purple-top tube).

### 2.1 Dried blood spots

Blood from neonates born in Israel is collected by a heel prick blotted on a 903 filter paper manufactured by Eastern Business Forms, United States. Recommended collection time is 36–48 h from birth.

### 2.2 Subjects

1,492,439 neonates were tested as part of the Israeli routine newborn screening panel**.**

### 2.3 Cutoff setting

Cutoff setting was as previously described by Staretz-Chacham et al. (2021), with the normal range for orotic acid set as <10 μmol/L and citrulline 10–50 μmol/L. Relevant here is the immediate referral of newborns with orotic acid equal to or above 10 μmol/L. Newborns with orotic acid equal to or above 10 μmol/L and citrulline within normal limits were referred as suggestive for hereditary orotic aciduria. Recall samples were requested only for initial results of orotic acid elevated above 4 μmol/L in combination with citrulline below 10 μmol/L.

### 2.4 Reagents

Stable-isotope labeled amino acids (NSK-A1), acyl-carnitines (NSK-B and NSK-B-G1), succinylacetone (CLM-6755) and orotic acid:H_2_O (1,3-15N2; NLM-1048) were from Cambridge Isotopes (Tewksbury, MA). HPLC-MS grade acetonitrile, methanol, and formic acid were from J.T. Baker, Fisher Scientific (Pittsburg, PA). Hydrazine hydrate was from Sigma-Aldrich (St. Louis, MO).

Quality control materials enriched with amino acids, acylcarnitines, and succinylacetone were from the CDC (Atlanta GA) and ClinChek RECIPE chemicals and instruments GmbH (Munich, Germany). Orotic acid (02750 Sigma Aldrich Israel, Rohovot, Israel) controls were prepared by serial dilution.

### 2.5 Sample extraction and analysis

Each DBS punch (3 mm) was placed in a well of a polypropylene U96-well plate (NUNC, Roskilde, Denmark) containing 100 μL of extraction medium (V/V: 80% acetonitrile, 20% DDW, 0.05% oxalic acid and 15 mM hydrazine), the plate was sealed with adhesive aluminum foil (Thermo Fisher Scientific, Rochester, NY) and extracted for 45 min at 45°C with shaking in a NCS incubator (Wallac, Turku, Finland). After extraction, 50 μL contents of each well were transferred to a new 96-well plate (350 µL Acquity collection plate, Waters Corporation Company, UK) containing 125 µL daily working solution (V/V: 80% acetonitrile, 20% DDW, 0.05% oxalic acid, and internal standards) and sealed by Cap-mat (7 mm round plug silicone/PTFE treated pre slit, Waters Corporation Company, United States). Orotic acid internal standard concentration per well was 7 µM. For analyses, samples were handled by Acquity H-class UPLC and the measurements by Xevo TQ-S micro (Waters Corporation Company) using flow-injection analysis by electrospray ionization tandem mass spectrometry. Mobile phase was 80% acetonitrile, 20% DDW and 0.02% formic acid. Tandem mass spectrometry analyses used multiple reaction monitoring (MRM) transitions. For orotic acid and orotic acid internal standard, MRM negative mode was used (parent 154.8, 156.8; daughter 111, 113; dwell 0.050, 0.10; collision V = 7; cone V = 25). Data processing and concentration determination were performed using MassLynx and NeoLynx (Waters Corporation Company). Results were interpreted by Specimen Gate software (Perkin Elmer, Turku, Finland).

### 2.6 Molecular diagnosis

The molecular whole exome sequencing were performed by CeGaT GmbH, Tübingen, Germany. No incidental findings were found, that according to the ACMG gene list and guidelines (Green et al., 2013).

#### 2.6.1 Prediction algorithms for pathogenicity analyses

The PolyPhen-2 and SIFT score predicts the possible impact of an amino acid substitution on the structure and function of a human protein. This score represents the probability that a substitution is damaging.

CADD, the third prediction program used, is a tool for scoring the deleteriousness of single nucleotide variants as well as insertion/deletions variants in the human genome (Rentzsch et al., 2019).

The human orotidine 5′-monophospahate decarboxylase 3D protein structure, NCBI, structure summary PDB ID: 3MW7, MMDB ID: 89666 was also used as prediction tool.

### 2.7 Metabolic confirmatory tests

Follow-up confirmatory tests included complete blood count, blood gas analysis, serum glucose and electrolytes, plasma lactate and ammonia, plasma amino acids and qualitative urinary organic acids.

## 3 Results

The routine Israeli newborn screening incorporates since 2014 the simultaneous measurement of both orotic acid and citrulline levels. Routine newborn screening of 1,492,439 neonates, including 328,337 Muslim Arabs, identified ten Muslim Arab newborns with elevated DBS orotic acid up to 10 times the upper reference limit, and with normal or borderline citrulline levels (Table 1). Newborns with orotic acid equal to or above 10 μmol/L and citrulline within normal limits were referred as suspected hereditary orotic aciduria. Four (three males) out of the 10 patients in our cohort had citrulline below 10 μmol/L and therefore were initially referred as suggestive for X-linked OTCD.

**TABLE 1:** Biochemical findings and UMPS genotype in hereditary orotic aciduria subjects identified by routine newborn screening.

No.	Citrulline normal 10–50 µM	Orotic normal <4.0 µM	1st ammonia µg/dL	Male/Female	*UMPS* variants^a^ NM_000373.4
1	18.5	31.8	120	M	c.24 G>C; p.L8F and c.342 T>G; p.N114K
2	19.6	15.9	not done	F	c.24 G>C; p.L8F and c.342 T>G; p.N114K
3	27.0	17.5	not done	M	c.24 G>C; p.L8F and c.342 T>G; p.N114K
4	9.9	13.6	Normal	M	c.24 G>C; p.L8F and c.342 T>G; p.N114K
5	10.2	20.0	Normal	M	c.24 G>C; p.L8F and c.342 T>G; p.N114K
6	9.5	25.0	110	M	c.24 G>C; p.L8F and c.342 T>G; p.N114K
7	13.5	45.1	Normal	M	c.24 G>C; p.L8F and c.342 T>G; p.N114K
8	9.5	17.1	not done	M	c.24 G>C; p.L8F and c.342 T>G; p.N114K
9	12.8	10.0	Normal	M	c.1132 G>C; p.A378P
10	8.5	9.8	Normal	F	c.1132 G>C; p.A378P

Newborns 1-6 are Bedouin Muslim Arabs from southern Israel, newborns 7–10 are Muslim Arabs from east Jerusalem. Newborns 5 + 6 and 7 + 8, and 9 + 10 are siblings.

^a^ All patients are homozygous for the listed change.

Confirmatory urine organic acids analysis demonstrated elevated urine orotate in all patients. All ten neonates (eight males and two females), harbor variants in the *UMPS* gene (Table 1). All patients have been followed up, except for one child with whom contact was lost, with the oldest child having now been followed for 6 years. None has developed megaloblastic anemia or neurologic sequelae, and all children have reached milestones appropriate for their age.

Among the 10 newborns diagnosed with hereditary orotic aciduria, six (patients 1-6 in Table 1) were Muslim Arabs belonging to a Bedouin community from the Negev region (southern Israel). Two of them (patients 5 + 6) were siblings, and all six were identified as double homozygotes for the c.24 G > C p.L8F and c.342 T > G p.N114K substitutions. The other four newborns (patients 7–10) were two pairs of Muslim Arabs siblings (not known whether they were of Bedouin descent or not), each pair of siblings originating from a different extended family in East Jerusalem. One pair (patients 7 + 8) shared the same double homozygous genotype found among the Negev Bedouins, while the other pair (patients 9 + 10) was homozygous for a c.1132 G > C p.A378P substitution.

**FIGURE 1:**
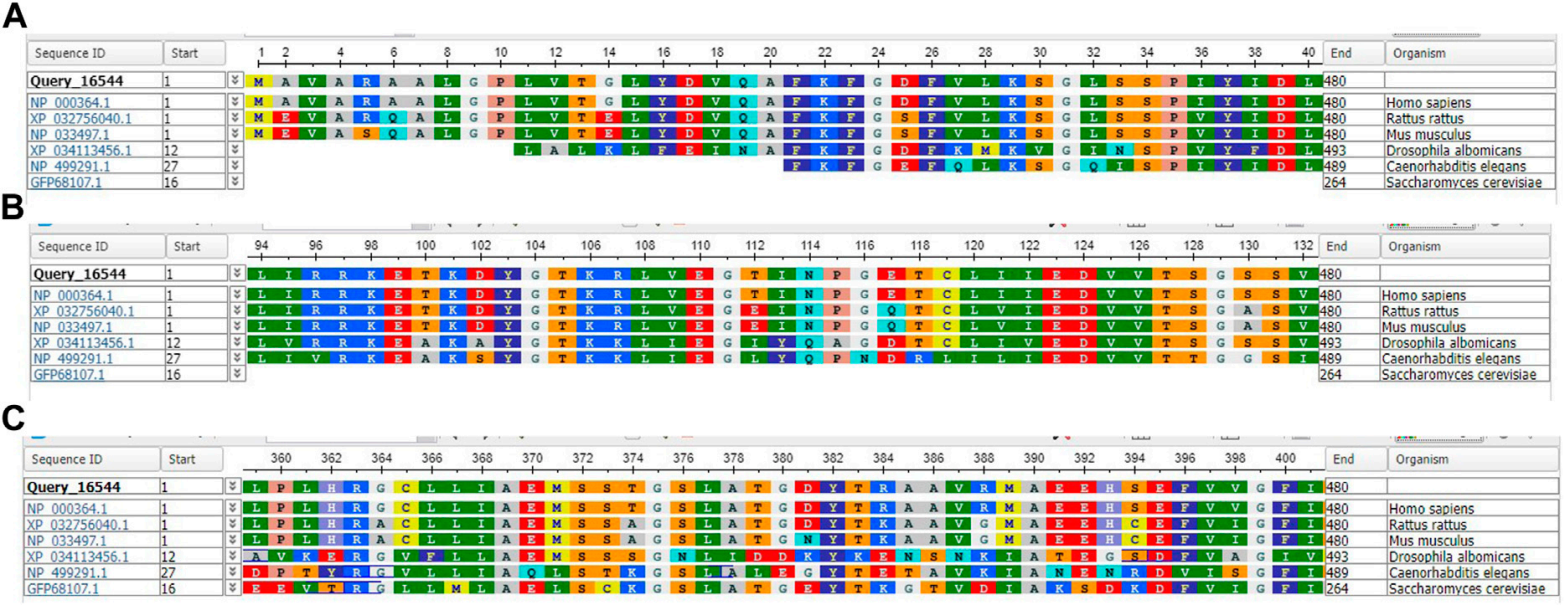
Standard Protein BLAST and alignment of uridine 5′-monophosphate synthase [*Homo sapiens*, *Rattus rattus*, *Mus musculus*, *Drosophila* albomicans] and Orotidine 5′-phosphate decarboxylase [*Caenorhabditis elegans*, *Saccharomyces cerevisiae*]. **(A)** Amino acid range 1–40 including 8 L. **(B)** Amino acid range 94–132 including 114 N **(C)** Amino acid range 359–401 including 378 A.

Both the p.L8F and the p.N114K amino acid substitutions are located in the N-terminal 214 amino acids region, harboring the OPRT activity. The p.A378P amino acid substitution is located in the C-terminal part harboring the ODC activity. The conservation of the two regions across species is described in Figure 1. Algorithms developed to predict the effect of missense changes on protein structure and function predicted the p.L8F variant to be disease-causing (SIFT: deleterious, PolyPhen-2: probably damaging, CADD score: 22.8) and all suggest that p.N114K variant is likely to be tolerated (Polyphen: benign, SIFT: tolerated, CADD score: 7.9) (Table 2). Both p.L8F and p.A378P variants are present twice in population databases (GnomAD allele frequency: 0.000007954, two heterozygous). Both parents of an affected child were found in segregation study to be heterozygous for their offspring’s p.L8F&p.N114K substitutions.

**TABLE 2:** In-silico variant impact prediction analyses.

	c.24 G>C; p.L8F	c.342 T>G; p.N114K	c.1132 G>C; p.A378P
^a^PolyPhen-2	possibly damaging	Benign	possibly damaging
Score	0.857	0.001	0.648
sensitivity	0.83	0.99	0.87
specificity	0.93	0.15	0.91
^b^SIFT	affects protein function	Tolerated	affects protein function
score	0.02	0.77	0.02
^c^CADD	Deleterious	Benign	Deleterious
score	22.8	7.9	26.4

^a^ PolyPhen-2 score ranges from 0.0 (tolerated) to 1.0 (deleterious). Variants with scores of 0.0 are predicted to be benign. Values closer to 1.0 are more confidently predicted to be deleterious. The score can be interpreted as follows: •0.0 to 0.15—Variants with scores in this range are predicted to be benign. •0.15 to 1.0—Variants with scores in this range are possibly damaging. •0.85 to 1.0—Variants with scores in this range are more confidently predicted to be damaging. (https://ionreporter.thermofisher.com/ionreporter/help/GUID-57A60D00-0654-4F80-A8F9-F6B6A48D0278.html).

^b^ SIFT scores ranges from 0.0 (deleterious) to 1.0 (tolerated) (https://ionreporter.thermofisher.com/ionreporter/help/GUID-57A60D00-0654-4F80-A8F9-F6B6A48D0278.html).

^c^ CADD is a tool for scoring the deleteriousness of single nucleotide variants as well as insertion/deletions variants in the human genome (Rentzsch et al., 2019).

The p.L8F and the p.N114K amino acid substitutions are in cis. The 3D protein structure (Supplementary Figure S2) indicates that the two amino acids do no interact with each other and therefore probably not causing the activity tolerance presences as asymptomatic patients.

## 4 Discussion

The purpose of newborn screening (NBS) is to identify newborns affected with diseases in which early diagnosis and prompt treatment will significantly change disease outcome (Jones and Bennett, 2002). Methionine and tyrosine as primary targets for core disorders in routine NBS have led to identification of secondary diagnoses such as hypermethioninemia (Couce et al., 2013) and transient tyrosinemia of the newborn (Adnan and Puranik, 2022). The addition of orotic acid analysis to our NBS Program in 2014 has been successful in the identification of a number of patients affected with urea cycle disorders (UCD) (Staretz-Chacham et al., 2021), as well as in identification of ten newborns with hereditary orotic aciduria. The introduction of expanded newborn screening has led to the identification of previously unrecognized, non-disease-causing variants of devastating disorders such as isovaleric acidemia (Ensenauer et al., 2004) and MCAD deficiency (Andresen et al., 2001). Herein, we report an analysis done in a cohort of patients identified by the NBS program with hereditary orotic aciduria based on increased orotic acid levels in DBS followed by confirmatory testing including urinary organic acids and molecular testing. To this day, no treatment has been administered to these patients, and all patients remain asymptomatic.

Hereditary orotic aciduria is an extremely rare condition with fewer than 30 cases reported in the literature. If left untreated, it may result in refractory megaloblastic anemia, neurodevelopmental disabilities, and crystalluria.

To the best of our knowledge, all individuals with hereditary orotic aciduria were reported to carry at least one missense variant allele, while no reports are available presenting affected individuals harboring bi-allelic null variants which are predicted to cause complete loss of UMPS protein function (Rogers et al., 1975; Wortmann et al., 2017). On the other hand, carrier individuals of null or missense variant may have persistent mild increase of urinary orotic acid secretion, lower than expected in OTC. Wortmann et al. reported 11 unrelated index cases referred for various signs and symptoms and 18 family members with mild and isolated orotic aciduria caused by heterozygous null or missense variants. The observed hypotonia and developmental delay in some of these individuals were thought to be due to ascertainment bias (Wortmann et al., 2017). Others reported heterozygote carriers of *UMPS* mutations with neurologic disabilities (Carpenter et al., 1997; Imaeda et al., 1998). The homodimeric structure of the UMPS protein might provide an explanation for the symptoms observed in heterozygote individuals, since the presence of a mutated allele may have a dominant negative effect on the wild-type allele, thereby reducing the functional homodimers to 25%. Here we report on 10 asymptomatic individuals harboring homozygous *UMPS* missense variants. Our newborn screening cutoff setting was as previously described by Staretz-Chacham et al. (2021), these sets of cutoffs will most likely result in avoiding the detection of heterozygous hereditary orotic aciduria carriers.

Homozygosity for a loss of function variant in the *umps* gene, p.R405x, in buffaloes and in the Holstein-Friesian breed cattle results in early embryonic death. Orotate level in the milk of this breed is four to 12 times normal (Schwenger et al., 1993; Sudhakar et al., 2021). Others reported that cows with partial deficiency of UMPS showed orotic acidemia and aciduria during lactation (Robinson et al., 1984; Shanks et al., 1984). In view of the above observations, it may be that a complete absence of this enzyme in humans is also incompatible with life, whereas variants causing a partial enzyme deficiency result in hereditary orotic aciduria either symptomatic or asymptomatic, as reported here.

The p.L8F & p.N114K double homozygous genotype has been identified in all six patients of the Bedouin Muslim Arabs from the Negev (patients 1-6 in Table 1), indicating that it is a possible founder allele in this population. The other four individuals are also Muslim Arabs from two extended families in East Jerusalem, one family with a pair of siblings sharing the same variant found among the Bedouins, and the other family harboring the p.A378P amino acid change. Both the p.L8F and the p.N114K substitutions are located in the N-terminal 214 amino acids portion of UMPS having OPRT activity. These variants have not been reported in the literature in individuals affected with UMPS-related conditions. Algorithms developed to predict the effect of missense changes on protein structure and function predicted the p.L8F variant to be disease-causing and all suggest that p.N114K variant is likely to be tolerated. This may indicate that the p.N114K variant is less significant in terms of its effect on protein function. One other option is that the linkage between the two changes causes the activity tolerance; however, the position of the two amino acids according to the 3D structure of the protein does not support such an interaction.

The p.A378P substitution, located in the C-terminal part with the ODC activity, is found in a more conserved sequence region across species (5 out of 6) including yeast, which may indicate its important function (Figure 1). It has not been reported in the literature in individuals affected with UMPS-related conditions. *In silico* analysis predicts this variant to be disease-causing. These predictions highlight the importance of the reported patients being asymptomatic, although the genetic and biochemical changes would have been suggestive for the known clinical disease.

### 4.1 Strengths and limitations of the study:

Although there is consistent follow-up of the patients, due to the patients' ages there is limited clinical follow-up. An additional study limitation is the limited genetic segregation analyses preformed, although in the study, two sets of affected siblings were followed and at least two sets of parents were found to be heterozygous. The study also lacks functional studies and detailed analysis of 3D protein structure.

## 5 Conclusion

To the best of our knowledge, this is the first report of asymptomatic individuals harboring homozygous *UMPS* mutations. This should raise consideration as to whether NBS for hereditary orotic aciduria as a secondary target warrants reporting, especially in populations without previously clinically identified patients. Asymptomatic or mild variants of hereditary orotic aciduria may be more common than previously recognized. Further identification and longer follow-up of such individuals will help to clarify this issue.

## Data Availability

The original contributions presented in the study are included in the article/Supplementary Material, further inquiries can be directed to the corresponding author.

## Supplementary material

Supplementary Material S1 The human orotidine 5′-monophospahate decarboxylase 3D location of the two amino acid involved in the cis amino acid alterations found in our patients. Amino acid leucine at position 8 is altered in our patients to phenylalanine (panel A) in combination with another cis alteration of asparagine at position 114 to lysine (panel B). NCBI, structure summary PDB ID: 3MW7, MMDB ID: 89666

## References

[B1] Adnan M. Puranik S. (2022). “Hypertyrosinemia,” in StatPearls [internet] (Treasure Island (FL): StatPearls Publishing). 2022 Jan–. PMID: 35201733. 35201733

[B2] Al Absi H. S. Sacharow S. Al Zein N. Al Shamsi A. Al Teneiji A. (2021). Hereditary orotic aciduria (HOA): A novel uridine-5-monophosphate synthase (*UMPS*) mutation. Mol. Genet. Metab. Rep. 26, 100703. PMID: 33489760; PMCID: PMC7807243. 10.1016/j.ymgmr.2020.100703 33489760 PMC7807243

[B3] Andresen B. S. Dobrowolski S. F. O'Reilly L. Muenzer J. McCandless S. E. Frazier D. M. (2001). Medium-chain acyl-CoA dehydrogenase (MCAD) mutations identified by MS/MS-based prospective screening of newborns differ from those observed in patients with clinical symptoms: Identification and characterization of a new, prevalent mutation that results in mild MCAD deficiency. Am. J. Hum. Genet. 68, 1408–1418. PMID: 11349232. 10.1086/320602 11349232 PMC1226127

[B4] Bailey C. J. (2009). Orotic aciduria and uridine monophosphate synthase: A reappraisal. J. Inherit. Metab. Dis. 32 (1), S227–S233. Epub 2009 Jun 27. PMID: 19562503. 10.1007/s10545-009-1176-y 19562503

[B5] Carpenter K. H. Potter M. Hammond J. W. Wilcken B. (1997). Benign persistent orotic aciduria and the possibility of misdiagnosis of ornithine carbamoyltransferase deficiency. J. Inherit. Metab. Dis. 20 (3), 354–358. PMID: 9266354. 10.1023/a:1005369726686 9266354

[B6] Couce M. L. Bóveda M. D. García-Jimémez C. Balmaseda E. Vives I. Castiñeiras D. E. (2013). Corrigendum to "Clinical and metabolic findings in patients with methionine adenosyltransferase I/III deficiency detected by newborn screening. Mol. Genet. Metab. 110 (3), 218–221. *PMID:* 23993429. 10.1016/j.ymgme.2015.01.011 23993429

[B7] Ensenauer R. Vockley J. Willard J. M. Huey J. C. Sass J. O. Edland S. D. (2004). A common mutation is associated with a mild, potentially asymptomatic phenotype in patients with isovaleric acidemia diagnosed by newborn screening. Am. J. Hum. Genet. 75 (6), 1136–1142. 10.1086/426318 15486829 PMC1182150

[B8] Fox R. M. Wood M. H. Royse-Smith D. O'Sullivan W. J. (1973). Hereditary orotic aciduria: types I and II. Am. J. Med. 55 (6), 791–798. PMID: 4753642. 10.1016/0002-9343(73)90260-x 4753642

[B9] Girot R. Hamet M. Perignon J. L. Guesnu M. Fox R. M. Cartier P. (1983). Cellular immune deficiency in two siblings with hereditary orotic aciduria. N. Engl. J. Med. 308 (12), 700–704. 10.1056/NEJM198303243081207 6828110

[B10] Green R. C. Berg J. S. Grody W. W. Kalia S. S. Korf B. R. Martin C. L. (2013). American College of Medical Genetics and Genomics. ACMG recommendations for reporting of incidental findings in clinical exome and genome sequencing. Genet. Med. 15 (7), 565–574. PMID: 23788249; PMCID: PMC3727274. 10.1038/gim.2013.73 23788249 PMC3727274

[B11] Huguley C. M. Bain J. A. Rivers S. L. Scogging R. B. (1959). Refractory megaloblastic anemia associated with excretion of orotic acid. Blood 14 (6), 615–634. PMID: 13651334. 10.1182/blood.v14.6.615.615 13651334

[B12] Imaeda M. Sumi S. Imaeda H. Suchi M. Kidouchi K. Togari H. (1998). Hereditary orotic aciduria heterozygotes accompanied with neurological symptoms. Tohoku J. Exp. Med. 185 (1), 67–70. 10.1620/tjem.185.67 9710947

[B13] Jones P. M. Bennett M. J. (2002). The changing face of newborn screening: Diagnosis of inborn errors of metabolism by tandem mass spectrometry. Clin. Chim. Acta 324 (1-2), 121–128. PMID: 12204433. 10.1016/s0009-8981(02)00238-3 12204433

[B14] Ma R. Ye J. Han J. Gao L. Wang C. Wang Y. (2022). Case report: A novel missense mutation c.517G>C in the *umps* gene associated with mild orotic aciduria. Front. Neurol. 13, 819116. PMID: 35356460; PMCID: PMC8959382. 10.3389/fneur.2022.819116 35356460 PMC8959382

[B15] McClard R. W. Black M. J. Livingstone L. R. Jones M. E. (1980). Isolation and initial characterization of the single polypeptide that synthesizes uridine 5'-monophosphate from orotate in Ehrlich ascites carcinoma. Purification by tandem affinity chromatography of uridine-5'-monophosphate synthase. Biochemistry 19 (20), 4699–4706. 10.1021/bi00561a024 6893554

[B16] Rentzsch P. Witten D. Cooper G. M. Shendure J. Kircher M. (2019). Cadd: Predicting the deleteriousness of variants throughout the human genome. Nucleic Acids Res. 47 (1), D886–D894. PMID: 30371827; PMCID: PMC6323892. 10.1093/nar/gky1016 30371827 PMC6323892

[B17] Robinson J. L. Dombrowski D. B. Clark J. H. Shanks R. D. (1984). Orotate in milk and urine of dairy cows with a partial deficiency of uridine monophosphate synthase. J. Dairy Sci. 67 (5), 1024–1029. PMID: 6547459. 10.3168/jds.S0022-0302(84)81401-0 6547459

[B18] Rogers L. E. Nicolaisen A. K. Holt J. G. (1975). Hereditary orotic aciduria: Results of a screening survey. J. Lab. Clin. Med. 85, 287–291. 1113015

[B19] Schwenger B. Schöber S. Simon D. (1993). DUMPS cattle carry a point mutation in the uridine monophosphate synthase gene. Genomics 16 (1), 241–244. 10.1006/geno.1993.1165 8486364

[B20] Shanks R. D. Dombrowski D. B. Harpestad G. W. Robinson J. L. (1984). Inheritance of UMP synthase in dairy cattle. J. Hered. 75 (5), 337–340. PMID: 6548235. 10.1093/oxfordjournals.jhered.a109951 6548235

[B21] Staretz-Chacham O. Daas S. Ulanovsky I. Blau A. Rostami N. Saraf-Levy T. (2021). The role of orotic acid measurement in routine newborn screening for urea cycle disorders. J. Inherit. Metab. Dis. 44 (3), 606–617. Epub 2020 Nov 30. PMID: 33190319. 10.1002/jimd.12331 33190319

[B22] Suchi M. Mizuno H. Kawai Y. Tsuboi T. Sumi S. Okajima K. (1997). Molecular cloning of the human UMP synthase gene and characterization of point mutations in two hereditary orotic aciduria families. Am. J. Hum. Genet. 60 (3), 525–539. PMID: 9042911; PMCID: PMC1712531. 9042911 PMC1712531

[B23] Sudhakar A. Khade A. Nayee N. Chandrasekhar Reddy R. V. Maurya B. (2021). Novel mutation in UMPS gene leads to false positive result of DUMPS (genetic disorder) in buffaloes: Need for gene sequencing before confirming results of RFLP in new species. J. Genet. 100, 55. PMID: 34344843. 10.1007/s12041-021-01305-2 34344843

[B24] Suttle D. P. Bugg B. Y. Winkler J. K. Kanalas J. J. (1988). Molecular cloning and nucleotide sequence for the complete coding region of human UMP synthase. Proc. Natl. Acad. Sci. U. S. A. 85 (6), 1754–1758. PMID: 3279416; PMCID: PMC279857. 10.1073/pnas.85.6.1754 3279416 PMC279857

[B25] Tubergen D. G. Krooth R. S. Heyn R. M. (1969). Hereditary orotic aciduria with normal growth and development. Am. J. Dis. Child. 118, 864–870. 10.1001/archpedi.1969.02100040866009 5353014

[B26] Wortmann S. B. Chen M. A. Colombo R. Pontoglio A. Alhaddad B. Botto L. D. (2017). Additional individual contributors. Mild orotic aciduria in UMPS heterozygotes: a metabolic finding without clinical consequences. J. Inherit. Metab. Dis. 40 (3), 423–431. Epub 2017 Feb 15. PMID: 28205048; PMCID: PMC5393157. 10.1007/s10545-017-0015-9 28205048 PMC5393157

