# Supplementary material for: Hereditary orotic aciduria identified by newborn screening
Source: Front Genet. 2023 Mar 14;14:1135267. doi: 10.3389/fgene.2023.1135267 (PMC10043439; doi:10.3389/fgene.2023.1135267)
Supplement: Supplementary file 1 [file DataSheet1.docx]

Figure 2: The human orotidine 5'-monophospahate decarboxylase 3D location of the two amino acid involved in the cis amino acid alterations found in our patients.

B

A


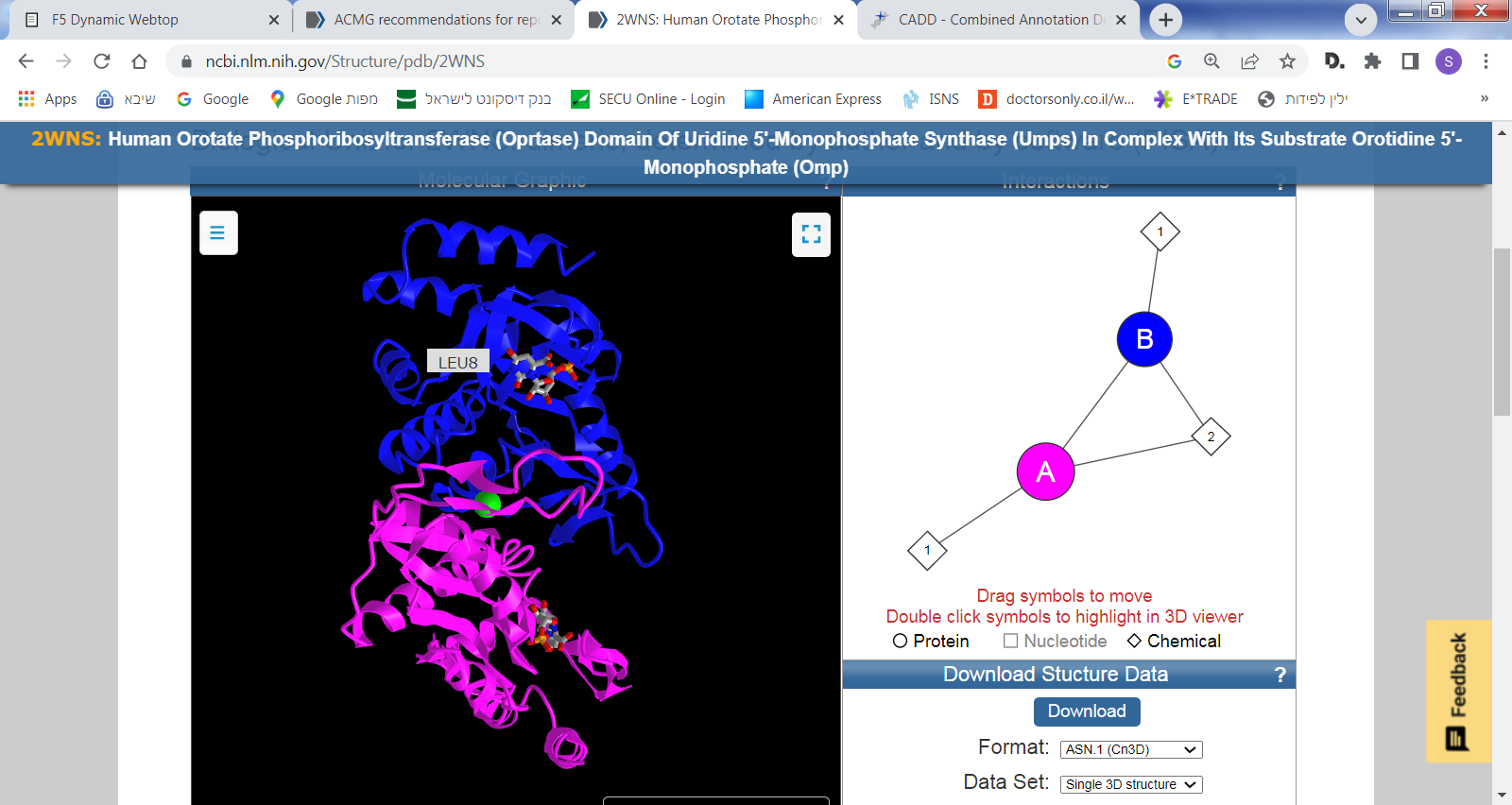

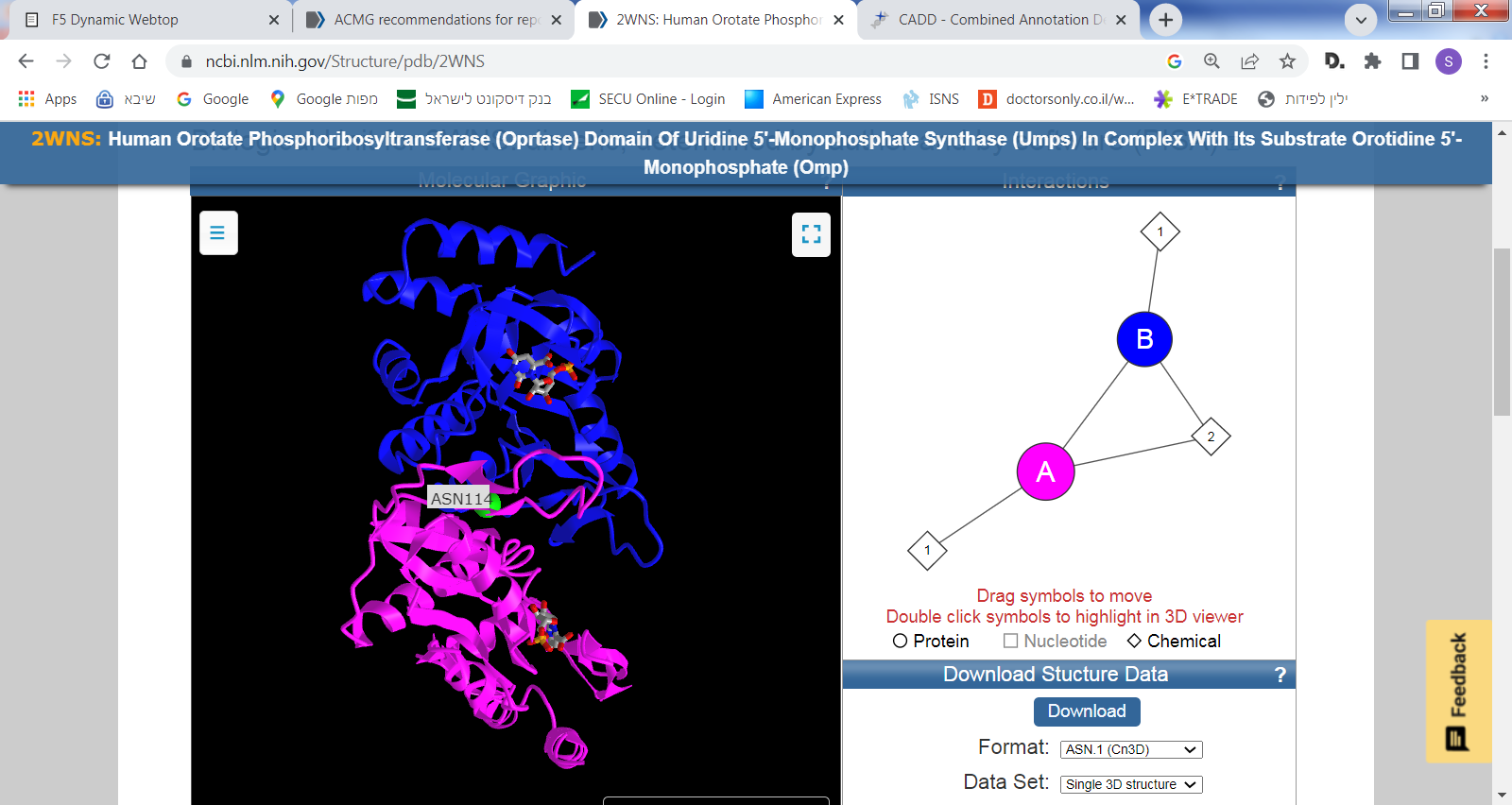


Amino acid leucine at position 8 is altered in our patients to phenylalanine (panel A) in combination with another cis alteration of asparagine at position 114 to lysine (panel B).

NCBI, structure summary PDB ID: 3MW7, MMDB ID: 89666
